# Food insecurity, dietary diversity and the right to adequate food among households in landslide-prone communities in Eastern Uganda: A cohort study

**DOI:** 10.1371/journal.pone.0283078

**Published:** 2023-04-13

**Authors:** Aziiza Nahalomo, Per Ole Iversen, Bård Anders Andreassen, Archileo Kaaya, Archangel Byaruhanga Rukooko, Peter Milton Rukundo

**Affiliations:** 1 Department of Nutrition, University of Oslo, Oslo, Norway; 2 Department of Haematology, Oslo University Hospital, Oslo, Norway; 3 Division of Human Nutrition, Stellenbosch University, Tygerberg, South Africa; 4 Norwegian Centre for Human Rights, University of Oslo, Oslo, Norway; 5 School of Food Technology, Nutrition and Bioengineering, Makerere University, Kampala, Uganda; 6 School of Liberal and Performing Arts, Makerere University, Kampala, Uganda; 7 Department of Nutritional Sciences and Dietetics, Kyambogo University, Kampala, Uganda; University of Naples Federico II: Universita degli Studi di Napoli Federico II, ITALY

## Abstract

We assessed food insecurity, dietary diversity and the right to adequate food among households in communities in Eastern Uganda that were affected by major landslides in 2010 and 2018. A prospective cohort study was applied to select 422 households during May-August (the food-plenty season) of 2019. In January-March (the food-poor season) of 2020, 388 households were re-assessed. Socio-demographic, food security, dietary diversity and right to adequate food data were collected using structured questionnaires. Four focus groups discussions and key informant interviews with 10 purposively sampled duty-bearers explored issues of food insecurity, dietary and the right to adequate food. The affected households had significantly higher mean (SE) food insecurity scores than controls, both during the food plenty season: 15.3 (0.5) vs. 10.8 (0.5), and during food-poor season: 15.9 (0.4) vs. 12.5 (0.0). The affected households had significantly lower mean (SE) dietary diversity scores than controls during the food plenty season: 5.4 (0.2) vs. 7.5 (0.2) and during the food poor season: 5.2 (0.2) vs. 7.3 (0.1). Multivariate analyses showed that the disaster event, education and main source of livelihood, were significantly associated with household food security and dietary diversity during the food-plenty season whereas during the food-poor season, the disaster event and education were associated with household food security and dietary diversity. During both food seasons, the majority of affected and control households reported to have consumed unsafe food. Cash-handout was the most preferred for ensuring the right to adequate food. Comprehension and awareness of human rights principles and state obligations were low. The severity of food-insecurity and dietary diversity differed significantly between the affected and control households during both food seasons. Moreover, the right to adequate food of landslide victims faced challenges to its realization. There is need for policy and planning frameworks that cater for seasonal variations, disaster effects and right to adequate food in order to reduce landslide victims’ vulnerability to food insecurity and poor dietary diversity. In the long-term, education and income diversification program interventions need to be integrated into disaster recovery programs since they are central in enhancing the resilience of rural livelihoods to shocks and stressors on the food system.

## Introduction

Ensuring food security for all is not only among the core aspect of the right to adequate food (RtAF), but also a priority goal under the United Nations (UN) Transforming our World: The 2030 Agenda for Sustainable Development [1, 2]. The UN Committee on Economic, Social and Cultural Rights clarified through its General Comment 12 (GC12) that the right to adequate food (RtAF) is realized *“when every man*, *woman and child*, *alone or in the community with others*, *have physical and economic access at all times to adequate food or means for its procurement”* [3]. All citizens are rights-holders whereas the State and other actors with State obligations and responsibilities are duty-bearers under international human rights law to which Uganda is a party. The RtAF not only compliments food security components with the State obligations of respect, protect and fulfil the right [3, 4], but also protects all humans to live in dignity, free from hunger, food insecurity and malnutrition [5, 6]. Moreover, the realization of the RtAF requires the recognition of the interdependency and progressive realization of all human rights. Also, the States have a core obligation to take the necessary action to mitigate and alleviate hunger, even in times of natural disasters [3].

The achievement of UN’s Sustainable Development Goal (SDG) number 2 on ending hunger and achieving food security by 2030, may be derailed. This is due to food insecurity and inequalities in access to food, unaffordability of healthy diets, climate change and natural disasters [7, 8]. Globally, in 2020, 811 million people were suffering from hunger and the number of moderate or severely food insecure people had risen from about 1.64 billion (22.6%) in 2014 to nearly 2.37 billion (30.4%) in 2020. Equally, more than 3 billion people could not afford a healthy diet in 2020. Notably, 290.9 million of the moderate or severely food insecure people live in Eastern Africa [7].

The RtAF and ensuring food security and nutrition for all, are recognized in the 1995 Uganda Constitution [9]. However, food insecurity has persisted in Uganda. By the end of 2020, 69.2% (30.6 million) Ugandans were food insecure among which 21.7% (9.6 million) were severely food insecure [7]. Similarly, 26% and 5% of households were already stressed and in a crisis of food insecurity, respectively [10], even before the Covid-19 effects had become apparent. The national average energy intake is at 8,715 kJ (2,083 kcal) per day per adult, below the recommended 9,210 kJ (2,200 kcal) [11]. Moreover, about 40% of Ugandans are estimated not to meet their energy requirements and the quality of Ugandan household’s diets is lacking with 40–60% of the energy intake derived from starchy staples [12]. Ugandans are also still grappling with malnutrition [13–15] and high poverty levels [16].

**Fig 1:**
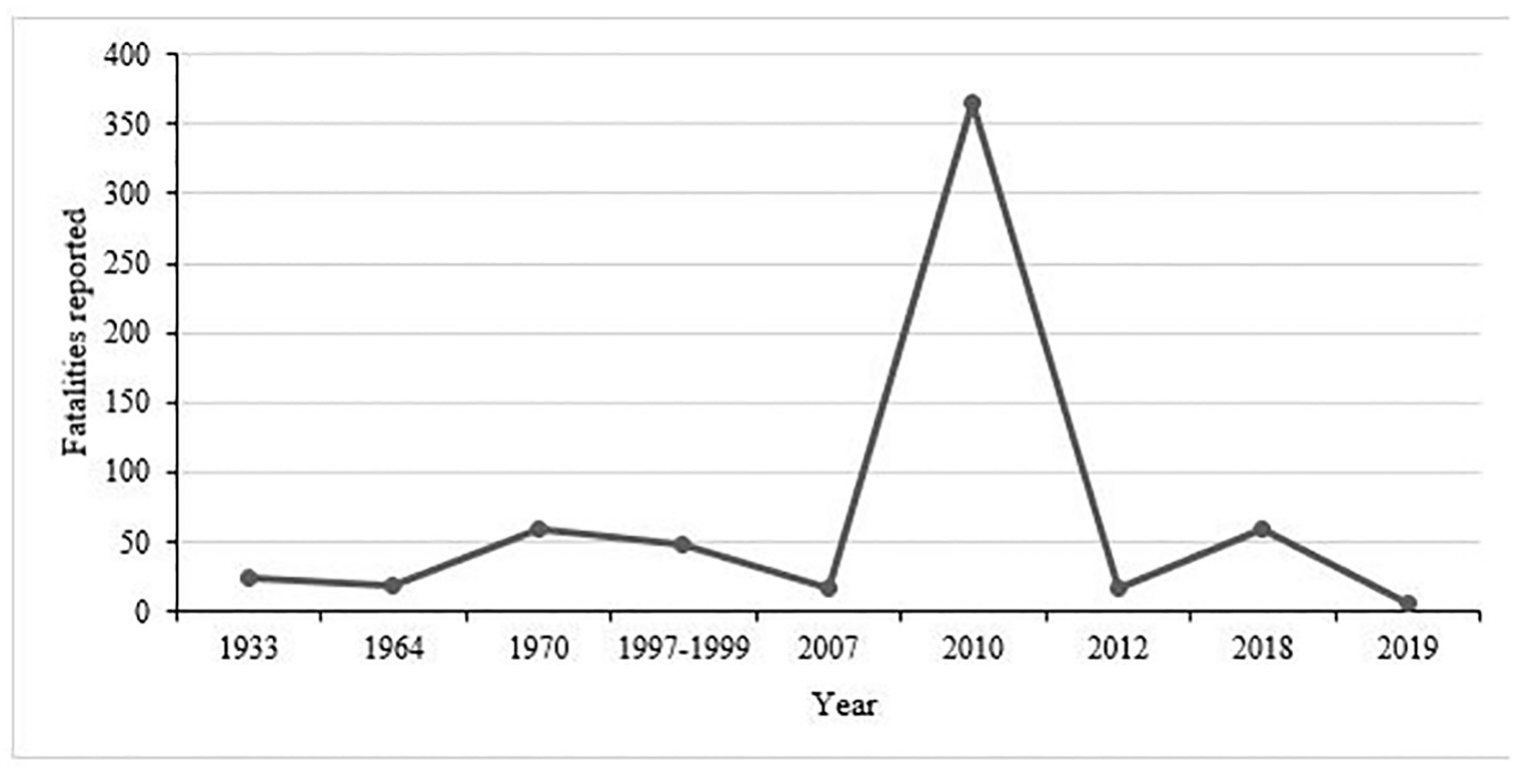
People killed by landslides in Bududa district of Eastern Uganda, 1900–2020. Data sources: [23–25, 27, 28].

Over the past years, Uganda has experienced frequent disasters such as landslides, floods and droughts, usually escalated by climate change [17, 18] (Table 1). The National Policy for Disaster Preparedness and Management acknowledges that on average, 200,000 Ugandans are affected annually by disasters [19]. During 2019–2020, excluding Covid-19 impacts, disaster events in Uganda affected nearly 800,000 people, displaced 21,000 families, and resulted in 152.2 million US dollars (USD) economic losses [20]. Morever, between 1900–2020, landslides were the second biggest killer among natural disasters in Uganda, causing an estimated death of 2,718 people [17] (Table 1). Among these, about 610 deaths occurred in Bududa District (Fig 1).

**Table 1:** Occurrence of key natural disasters in Uganda, 1900–2020.

Natural disaster	Total deaths	Total number of people affected	Total damage (‘000 USD)	References
Drought	194	4,975,000	1,800	[18, 26]
Floods	343	1,060,559	6,871	[18, 26]
Epidemics	3,670	345,701	Not known	[18, 26]
Landslides	2,718	151,546	Not known	[17, 18, 26, 27, 28]
Storm	23	47	Not known	[18, 26]
Earthquake	115	58,100	71,500	[18, 26]

Natural disasters limit peoples’ access to adequate food through interference with the food security components via destruction of the food systems and livelihood-related infrastructure [21]. This may result in malnutrition and hunger predominantly in areas where chronic food insecurity is already a significant problem and thus create vicious cycles of poverty, disease and hunger [2]. Consequently, the achievement of the right to adequate food [5, 22] and SDG targets 2.1 and 2.2 related to food security and nutrition [1], are disrupted.

Bududa District in Eastern Uganda has experienced several devastating landslides with the earliest records dating to as early as 1933 (Fig 1), with catastrophic effects to life, property, crops, livestock, infrastructures and the environment [23]. Unfortunately, the economic damage from these landslides is not well documented [24]. In March 2010, a major landslide in Bukalasi sub-county in Bududa District left over 360 dead, thousands displaced and infrastructures, food crops and livestock destroyed [23]. In October 2018, another major incident occurred in the same sub-county and left 60 dead, 858 people displaced and 144 houses destroyed [25].

As a result of the major 2010 landslide, we performed a cross-sectional study and identified lower food insecurity, higher dietary diversity and food variety scores among the affected communities compared to the unaffected (control) communities in Bududa District [29]. Food varieties were also higher among farmers and relief food recipients compared to the non-farmers and non-relief food recipients. Still, the affected households had a higher likelihood to skip a day without eating a household meal compared to the control households [30]. However, there is limited longitudinal cohort data on how landslide disaster affect household food security, dietary and the RtAF among victims of landslides in the country. Yet, such data are very important in the country’s efforts to plan for these vulnerable categories of people. Hence, in this follow-up study we aimed to assess food insecurity, dietary diversity and the RtAF among households in the landslide-prone communities of the 2010 and 2018 landslide disasters in the Bududa District.

## Materials and methods

### Study design and setting

A prospective cohort study was performed in the periods May-August 2019 and January-March 2020 and we report the results according to the STROBE guidelines [31]. The study site was the Bududa District in the Bukalasi sub-county, which was devastated by the landslides of 2010 and 2018. The neighboring sub-county Bubiita acted as the control. Bududa District is located on the foot of the South-Western slopes of Mount Elgon, about 250 km from Kampala, Uganda’s capital city. The district’s elevated topography subjects Mount Elgon region to regular disastrous floods and landslides [32]. The average precipitation of the area is above 1500 mm of rainfall per year [23]. The district’s population is 210,173 people [33], with a high population density of about 952 persons per km^2^. The continued agricultural activities on the steep slopes of Mount Elgon with V-shaped valleys and river incisions precipitate a high risk for landslides [23]. The majority of the population is rural and relies mainly on subsistence agriculture [23, 33].

Bukalasi sub-county is located on the steep slopes of Mt. Elgon with loose soil types, bi- modal rainfall patterns, high population growth rate and increased land cultivation making it more vulnerable to landslides and related consequences [34]. The natives are mainly rural subsistence farmers and the steep terrain limits their accessibility to the markets [23].

Bubiita sub-county is situated on the low terrain at the foot of Mt. Elgon with fertile soils and bi-modal rainfall patterns. It has a high population growth rate, however it less vulnerable to landslides and their consequences due to its location on the low terrain [34]. The natives are mainly subsistence farmers and a small portion of traders with adequate access to the market. The population is rural with a small semi-urban segment [35].

### Study participants

Study participants were household heads in the study area, focus group discussants (FGD) and key informants (KIs).

The FGDs constituted adult women and men who were members of the local council at village and parish level in the study area whereas KIs constituted individuals or representatives from the Bududa District and relevant government departments. Specifically, they were: the Chairperson Disaster Management Committee, Bududa hospital nutritionist, Senior Environmental Officer, Health Inspector, Community Development Officer, Production Officer, Sub-county Chiefs and Local Council Leaders.

### Sample size

This study is part of a research project that involved a cohort and descriptive survey among the 2010 and 2018 victims of landslide disasters in Eastern Uganda. A computed sample size of 418 households was targeted based on the 35.9% stunting level reported in children 6–59 months old in the Bugisu sub-region [36], due to the absence of reliable effect measures of landslides on food insecurity and dietary diversity. Details for sample size and sampling procedure of households are reported in our previous study [13].

Participants for FGDs in each sub-county were sampled independently from households which were not selected for quantitative interviews. Four FGDs were targeted, two from the affected and two from the control sub-county. Six to ten participants for each FGD were targeted. The leadership in each sub-county assisted to mobilize the FGD participants.

Ten key informants were purposively selected on the basis that they were conversant with the subject matter being studied or were in positions of authority in their respective institutions or ministries in areas related to landslides, food security, diet and the right to adequate food.

### Study approvals

The Uganda National Council for Science and Technology (UNCST) (no: SS 4967), Makerere School of Health Sciences Research Ethics Committee (no: 2018–082) and the Norwegian Regional Committee for Medical and Health Research Ethics (no: 2019/917) approved this study. Participation into the study was by informed and voluntary written or thumb printed consent.

### Data collection and measurements

The research applied mixed methods, with a combination of quantitative and qualitative research activities suited to an interdisciplinary exploration of food security, dietary and the RtAF [37]. Quantitative data from household heads were collected twice: (i) in the food-plenty season (May-August 2019), and (ii) after six months at food-poor season (January-March 2020) to account for variations in food-plenty and food-poor seasons. Trained research assistants with at least a College or University level of education collected the quantitative data from the household heads. This was through face-to-face interviews using pretested and structured questionnaires that were translated from English to the local language (Lumasaba) and back-translated into English. The questionnaire included mainly close-ended questions related to demographic and socio-economic information, experiences on access to food, the frequency and diversity of food groups consumed and the RtAF.

Qualitative data from KIs and FGDs were collected once during the food-poor season (January-March of 2020) using semi-structured interviews and discussion guides, respectively, in a face-to-face set up. The aim was to get a broader understanding of the food security, dietary and the RtAF in the study area. Both written and audio records were collected with permission of the participants.

### Household food insecurity

Household food insecurity was assessed using standardized food access and hunger scales adapted from a combination of the Household Food Insecurity Access Scale (HFIAS) index [38] and the Community Childhood Hunger Identification Project (CCHIP) scale index [39, 40]. Importantly, CCHIP provides a more understanding of the effects of food insecurity on household members by accounting for child hunger [39, 40]. Additionally, the scoring of CCHIP is similar to HFIAS, and the two tools provide a measure to understand the food insecurity problem in resource-limited settings, especially among rural populations that rely mainly on subsistence farming [41].

The combined HFIAS and CCHIP scale has eleven food-insecurity experience-based indicators related to worry about lack of food, insufficient quality and quantity of meals, and going to sleep hungry, both in adults and children of the household in the last 30-days preceding the survey. The indicators included: (1) having skipped a day without a general household meal of breakfast, lunch or supper; (2) children ever went to bed hungry because of lack of food; (3) children were allowed to roam and eat elsewhere because of lack of food; (4) sought financial support to buy food; (5) children having eaten less food because of there not being enough food; (6) sought food assistance from neighbors, relatives and friends; (7) limited portion sizes at meals because of there not being enough food; (8) reduced food for adults because of there not being enough food; (9) parents eating less because of there not being enough food; (10) purchased food on credit; and (11) relied on less-preferred, less-expensive food.

For each item, the respondent selected a frequency of the experience as: never, rarely, sometimes, or always. Never was scored as 0; a frequency of one to two times was considered as ‘rare’ and scored 1 point; three to ten times was considered as ‘sometimes’ and scored 2 points; and more than ten times was considered as ‘often’ and scored 3 points [38, 39]. If the household’s response to all the eleven questions was often reported ‘yes’, a maximum score of 33 points was given and a minimum score of 0 if the respondent answered ‘never’ to all the questions. The generated score from 0 to 33 reflected a single statistical dimension of food insecurity. A score of 0 indicated food secure while a score between 1–33 indicated food insecure, i.e. the higher the score, the more the households experienced food insecurity.

### Household dietary diversity

Household dietary diversity was assessed using the Household Dietary Diversity Score (HDDS) to establish each household’s access to different types of food. This was based on a retrospective recall by the household’s head about the frequency of the household eating food items listed in a food frequency questionnaire (FFQ). This FFQ was adapted for Uganda and contained commonly eaten foods (n = 86) grouped into 12 groups: (1) cereals (2) legumes, (3) starchy roots, tubers and plantain, (4) vegetables, (5) fruits and fruit juice, (6) meat and meat products, (7) poultry and eggs, (8) milk and milk products, (9) fish, (10) fats and oils, (11) sugars and confectionaries, and (12) condiments, spices and beverages [42]. The HDDS is a continuous score which measures the consumption of these 12 food groups within the past 24 hours. Household heads were asked whether the household had eaten each of the listed food items in the previous 24 hours and the approximate frequency of use of each of the eaten items. The information regarding food items consumed in the household over the 24 hours preceding the interview was used to compute the HDDS.

The HDDS was calculated by summing the number of food groups consumed by each respondent over the previous 24-hour period. Minimum score was 0 if the household did not consume any food group and the maximum score was 12 if the household consumed all the food groups. This score was used as a proxy to estimate the dietary quality given their suitability in resource limited settings. The higher the score was, the higher was the nutrient adequacy of the diets consumed while the lower the score, was the lower the dietary nutrient adequacy.

### Perceptions on the right to adequate food, food and nutrition security and diet diversity

Perceptions about the right to adequate food, food and nutrition security and diet diversity were assessed based on questions adapted and modified from the “Guide to conducting right to food assessment” by FAO [43], because the right to food encompasses food security attributes including nutrition security and diet [3]. A pre-coded and structured questionnaire with mainly closed-ended questions regarding perceptions on the right to adequate food, food and nutrition security and diet diversity during disaster in Bududa District, was used for data collection from household heads. Questions included: (1) whether in the past 30 days there were instances when: (a) a household did not have sufficient food for more than 2 days, (b) a household head felt the household was not eating food that was safe, (c) a household head felt the household was eating less nutritious food and could not do much about it; (2) whether providing food for the household limited the household’s ability to provide other amenities like health, water, housing, clothing and education; (3) whether the landslides had affected the household’s food and nutrition security and the RtAF; (4) awareness about the principles of human rights of participation, accountability, non-discrimination and transparency; (5) awareness about the State obligations of respect, protect and fulfill; and (6) the preferred means to ensure the right to adequate food of landslide disaster victims.

Using discussion and interview guides, FGDs and KIIs were held to get the broader perspective on food security, diet and the RtAF. Guiding questions included: What is the situation of food and nutrition security in the study area; where, when and who are the most affected and why; whether landslides affected the food and nutrition security and the RtAF of landslides victims; whether the disaster response in the study area is satisfactory; whether the human rights principles of participation, accountability, non-discrimination and transparency are taken into consideration during the response of public authorities to the disasters; the perception on the obligation of the State to ensure that no Ugandan suffers from hunger and malnutrition even in times of disaster; how the State should ensure the realization of the RtAF of landslide disaster prone communities; and the preferred means to ensure the RtAF of disaster victims.

The FGDs were conducted at the respective sub-county headquarters. A facilitator fluent in both English and the local language led the FGDs and the FGD participants were told beforehand to be at liberty to discuss in English or their native languages, and that all answers were equally important. The discussions ranged from 60–90 minutes. Interviews with KIs were conducted in English on appointment by the first author (A.N) and took place in the participant’s office. The interviews ranged from 45–90 minutes. Both audio- and written data were collected during the FGDs and KIIs. Written informed consent to participate and record the interview/discussion was sought from each participant prior to the start of each session.

### Statistical analyses

Analyses for quantitative data were conducted using Stata version 16.1 statistical software [44]. The Levene’s independent samples t-test tested the unadjusted mean differences in the household and dietary diversity scores because of its appropriateness for application to both normally and non-normally distributed data. The two dependent outcomes of food insecurity and dietary diversity scores were first tested for linearity with each other using Pearson’s correlation (r). Given that the two dependent variables showed a small positive correlation (r = 0.08) in the food-plenty season and a small negative correlation (r = -0.27) in the food-poor season, a one-way analysis of covariance (ANCOVA) and multivariate analysis of covariance (MANCOVA) models were used to test for univariate and multivariate effects while adjusting for the disaster effect and socio-demographic covariates. The covariates included were: interviewed household head, age of the household head, education level of household head, family size, main source of livelihood, household ownership of assets or entitlements and migration of a household member in the past 12 months preceding the survey.

Responses from household heads regarding perceptions on food and nutrition security, diet and the RtAF were treated as categorical variables in the analysis. Pearson chi-square test was used to examine associations between these categorical variables, using a p < 0.05 as a level of significance.

Data from KIs and FDGs were triangulated to augment the quantitative data outcomes using thematic analysis. The process involved transcription of translated information which was also cross-checked to ensure quality, followed by identification and coding of key words and phrases with similar impressions. The coded information was assigned into groups and categorized into themes. The generated themes were reviewed to ensure that the themes were accurate representations of the data. Defining and renaming of the generated themes was then done to establish a sequence of patterns and associations related to study themes and included in the results and discussion of results accordingly.

### Inclusivity in global research

Additional information regarding the ethical, cultural, and scientific considerations specific to inclusivity in global research is included in the S1 Checklist.

## Results

### Characteristics of the study population

**Fig 2:**
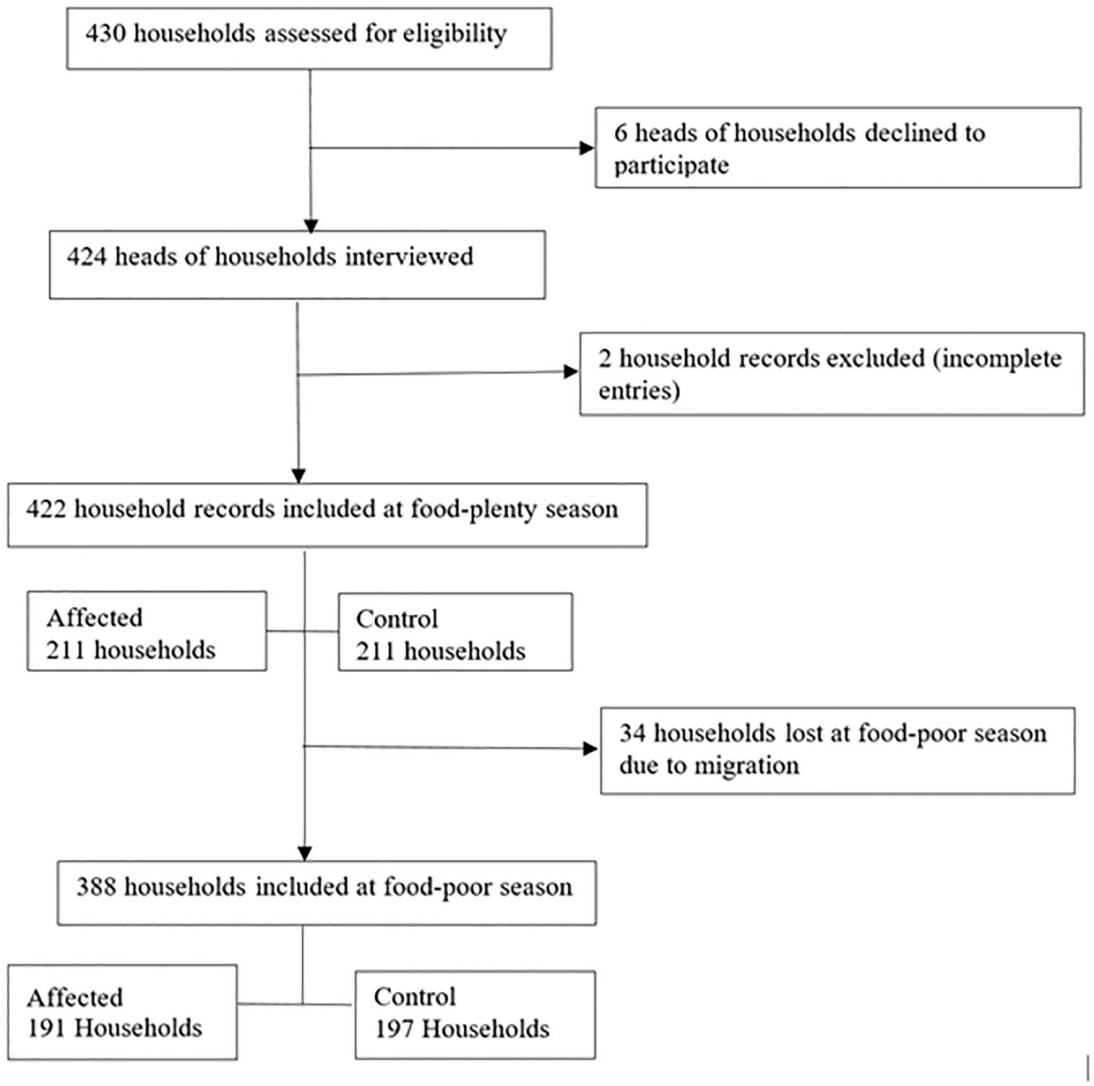
Flow chart showing enrollment of study participants into the study.

A total of 422 households participated in the study during the food-plenty season while 388 households were followed-up during the food-poor season (Fig 2). Thirty-six participants in four focus groups and 10 key informants participated in the study.

There were more maternal household heads from the affected than the control group being interviewed during the food-plenty season (p = 0.003), but not during the food-poor season (Table 2).

**Table 2:** Characteristics of the participating households^a^.

	Food-plenty season (n = 422)			Food-poor season (n = 388)		
Variables	Affected (n = 211)	Control (n = 211)	P-value^b^	Affected (n = 191)	Control (n = 197)	P-value^c^
Interviewed household head						
Father	40 (18.9)	17 (8.1)	0.003*	134 (70.2)	157 (79.7)	0.11
Mother	161 (76.3)	174 (82.5)		40 (20.9)	25 (12.7)	
Grandparents or elderly siblings	10 (4.8)	20 (9.5)		17 (8.9)	15 (7.6)	
Age of the household head (years)	32.1 ± 11.7	32.3 ± 11.5	0.71	33.2 ± 11.9	33.9 ± 11.8	0.56
Education level of household head						
None	14 (6.7)	13 (6.2)	0.18	6 (3.1)	18 (9.1)	0.21
Primary	156 (73.9)	145 (68.7)		150 (78.6)	142 (72.1)	
Secondary	39 (18.5)	47 (22.3)		33 (17.3)	32 (16.2)	
≥ College	2 (0.9)	6 (2.8)		2 (1.0)	5 (2.5)	
Household size	6.5 ± 2.6	5.9 ± 2.3	0.014*	6.6 ± 2.6	6.3 ± 2.3	0.16
Main source of livelihood						
Farming	174 (82.5)	125 (59.2)	0.000*	178 (93.2)	173 (87.8)	0.004*
Trading	17 (8.1)	18 (8.5)		4 (2.1)	13 (6.6)	
Casual laborer	16 (7.6)	44 (20.9)		9 (4.7)	7 (3.6)	
Fishing or wage employee	4 (1.8)	24 (11.4)		0 (0.0)	4 (2.0)	
Main source of food						
Own production	150 (71.1)	80 (37.9)	0.000*	100 (52.4)	61 (30.9)	0.000*
Purchase	33 (15.6)	121 (57.3)		90 (47.1)	133 (67.6)	
Own labor	28 (13.3)	10 (4.7)		1 (0.5)	3 (1.5)	
Lost any household members in the past 12 months preceding the survey						
Yes	32 (15.2)	38 (18.0)	0.56	8 (4.2)	17 (8.6)	0.07
No	179 (84.8)	173 (81.9)		183 (95.8)	180 (91.4)	
Migration of any member of the household in the past 12 months preceding the survey						
Yes	19 (9.0)	54 (25.6)	0.000*	38 (19.9)	16 (8.1)	0.001*
No	192 (91.0)	157 (74.4)		153 (80.1)	181 (91.9)	
Household ownership of assets or entitlements^d^						
Yes	137 (64.9)	143 (67.8)	0.21	57 (29.8)	121 (61.4)	0.000*
No	74 (35.1)	68 (32.2)		134 (70.2)	76 (38.6)	
Number of meals consumed/day	2.2 ± 0.6	2.2 ± 0.7	0.07	2.3 ± 0.6	2.3 ± 0.6	0.07
Food insecurity scores (FIS)	15.3 ± 6.8	10.8 ± 5.1	0.000*	15.9 ± 7.0	12.5 ± 6.5	0.000*
Dietary diversity scores (DDS)	5.4 ± 2.6	7.5 ± 2.2	0.000*	5.2 ± 2.5	7.3 ± 2.6	0.000*

^a^Values are numbers (%) or means ± standard deviation.

^b^P-value is for chi square or t test between affected and controls during food-plenty season.

^c^P-value is for chi square or t test between affected and controls during food-poor season.

^d^Such as farm, livestock, poultry, motorcycle, bicycle.

*Denotes statistical significance when p < 0.05.

Primary level was the most attained education level among both the affected and the control households during both food seasons. Moreover, farming was the main source of livelihood, but was different between the affected and the control during both the food-plenty (p < 0.001) and the food-poor season (p = 0.04). Additionally, migration of any household member in the past 12 months preceding the study was significantly lower in the affected compared with the control households during the food-plenty season. However, it increased significantly among the affected compared to the controls during the food-poor season. Household ownership of assets was higher during the food-plenty compared to the food-poor season in both the affected and the control households. Conversely, it decreased during the food-poor season among the affected compared to the control households (p < 0.001) (Table 2).

### Household food insecurity

Overall, the mean household food insecurity scores were significantly higher among the affected compared to the controls during both food seasons (Table 3). Moreover, FGD participants and KIs further cited that the affected communities faced more food insecurity compared to their counterparts and the situation was worse during the food-poor season. Lack of enough to eat and to feed the young children stood out as a major issue (S1 Table).

**Table 3:** Adjusted differences in household food insecurity and dietary diversity scores.

	Food-plenty season (n = 422)								Food-poor season (n = 388)							
	ANCOVA								ANCOVA							
Variables		Food insecurity^a^			Dietary diversity^b^			MANCOVA^c^		Food insecurity^a^			Dietary diversity^b^			MANCOVA^c^
	n	Mean	SE	P	Mean	SE	P	P	n	Mean	SE	P	Mean	SE	P	P
Disaster																
Affected	211	15.3	0.5	<0.001*	5.4	0.2	<0.001*	<0.001*	191	15.9	0.4	<0.001*	5.2	0.2	<0.001*	<0.001*
Control	211	10.8	0.5		7.5	0.2			197	12.5	0.4		7.3	0.2		
Interviewed household head																
Fathers	57	12.9	1.0	0.59	6.6	0.3	0.48	0.06	291	14.2	0.3	0.38	7.8	0.1	0.25	0.25
Mothers	335	13.3	0.4		6.9	0.1			65	13.6	0.8		8.0	0.2		
Others^d^	30	10.4	1.4		7.5	0.4			32	14.8	1.1		7.3	0.3		
Education level of the household head																
≤ primary	327	15.5	0.4	<0.001*	5.7	0.1	<0.001	<0.001*	305	13.6	0.7	0.19	5.5	0.1	<0.001*	<0.001*
≥ secondary	95	13.3	0.8		7.7	0.2			83	14.3	0.3		8.8	0.2		
Household size																
≤ 5 members	195	12.9	0.6	0.253	7.1	0.2	0.87	0.52	159	13.2	0.5	0.044	7.6	0.2	0.23	0.42
≥ 6 members	227	13.1	0.5		6.9	0.2			229	14.9	0.4		7.8	0.1		
Main source of livelihood																
Farming	299	11.9	0.4	<0.001*	6.5	0.1	0.015	<0.001*	351	14.1	0.3	0.08	7.7	0.1	0.93	0.98
Others^e^	123	15.8	0.6		7.1	0.2			37	15.8	1.1		8.1	0.3		
Household ownership of assets or entitlements^f^																
Yes	282	12.6	0.4	0.07	7.0	0.1	0.56	0.17	178	12.3	0.4	<0.001*	8.0	0.2	0.63	0.69
No	140	13.8	0.6		8.6	0.2			210	16.5	0.4		7.6	0.1		

^a^Adjusting for disaster effect, interviewed household head, household head’s education level, family size, main source of livelihood, household ownership of assets or entitlements, migration of any household member in the past 12 months preceding the survey and household dietary diversity score.

^b^Adjusting for disaster effect, interviewed household head, household head’s education level, family size, main source of livelihood, household ownership of assets or entitlements, migration of any household member in the past 12 months preceding the survey and household food insecurity score.

^c^Test for multivariate effect of each variable on both outcomes after adjusting for covariates. Given two dependent variables in the model, Hotelling’s Trace value is reported.

^d^Refers to grandparents or elderly siblings,

^e^such as trading, wages, carpentry,

^f^ such as farm, livestock, poultry, motorcycle, bicycle.

*Denotes statistical significance when p < 0.05.

### Household dietary diversity

**Fig 3:**
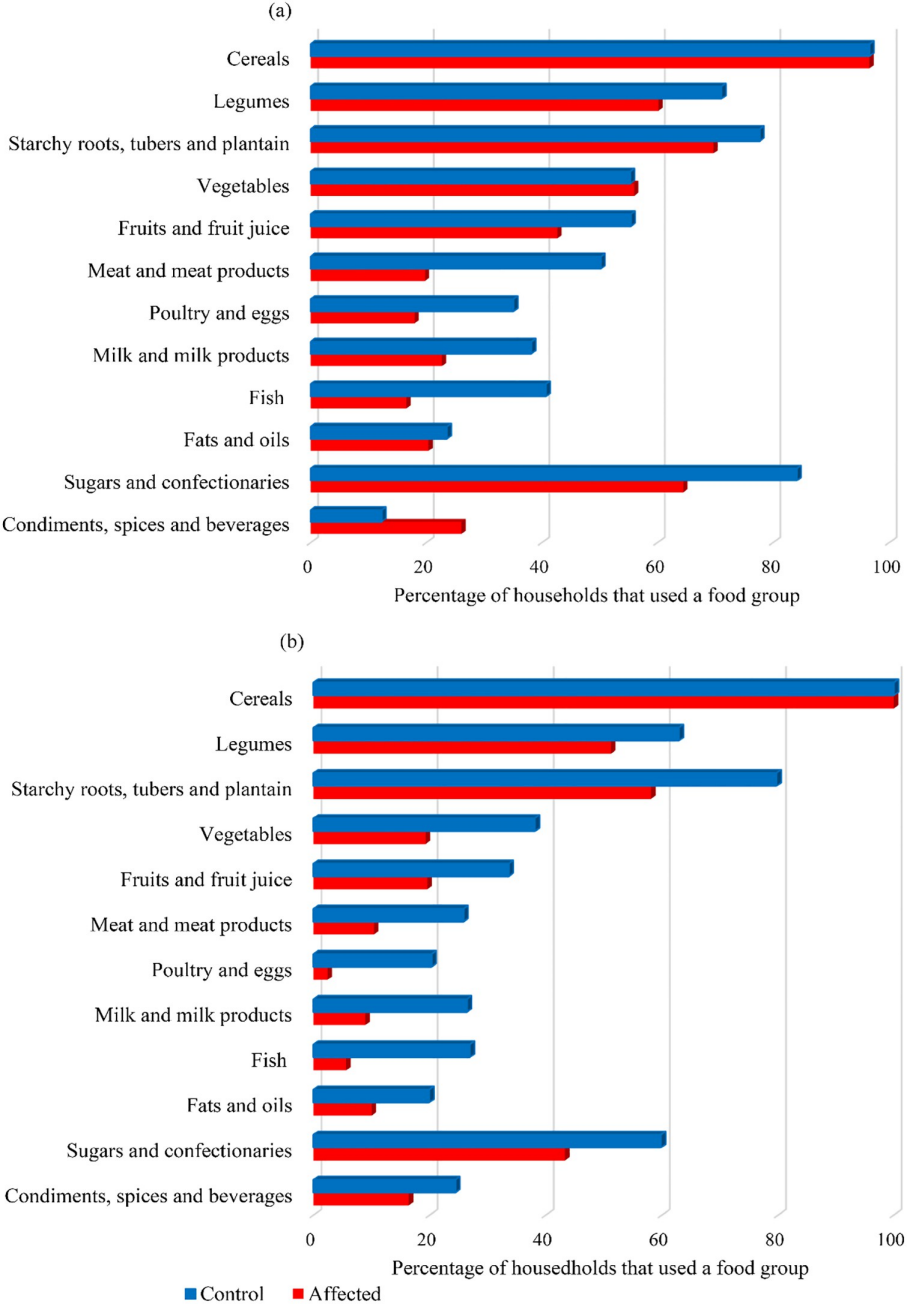
Food groups consumed over the 24 hours period by households in the landslide-prone communities during: (a) food-plenty season (May-August) and (b) food-poor season (January-March).

The affected households exhibited significantly lower household dietary diversity scores during both the food-plenty and the food-poor seasons compared with the controls (Table 3). Moreover, cereal-based foods, legumes, starchy roots, tubers and plantain and sugars and confectionaries were the most consumed food groups during both food seasons by both the affected and control households (Fig 3). Consumption of animal-source foods was very low among the affected compared to the controls and significantly decreased during the food-poor season. Intake of food of lower dietary diversity among the affected communities was also noted by majority of the KIs and FGDs (S1 Table).

### Multivariate effects on food security and dietary diversity

After controlling for potential covariates, ANCOVA results indicated that the disaster event, education level and main source of livelihood were associated with food insecurity at food-plenty season (p < 0.001 in all) whereas the disaster event and household ownership of assets or entitlements were associated with food-insecurity (p < 0.001 in both) during the food-poor season (Table 3). Furthermore, ANCOVA results indicated that the disaster event and education level were associated with poor dietary diversity during both food-seasons (p < 0.001 in both) (Table 3).

The MANCOVA findings showed that the disaster event, education level and main source of livelihood were associated with both household food insecurity and dietary diversity at food-plenty season (p < 0.001 in all) whereas during the food-poor season, the disaster event and education level were associated with both outcomes (p < 0.001 in both) (Table 3).

### Perceptions on the right to adequate food, food and nutrition security and diet diversity

The household’s perceptions about food and nutrition security, diet and the right to adequate food differed significantly between the affected households and the controls during both food seasons (Table 4). Regarding the question of a household not consuming safe food, there were significant differences in the responses between the affected and controls during both food seasons. The majority (81.6%) of the affected compared to 68.2% of the control during the food-plenty and 91% of the affected compared to 65% of the controls during the food-poor season, reported that they were consuming food that was not safe, but they could not do much about it (Table 4). This was consistent with information from KIs who linked intake of non-safe food e.g. maize flour and beans which were insect-infested and with a bad smell and taste, due to lack of enough food and money throughout the food seasons (S1 Table).

**Table 4:** Households’ perceptions about food and nutrition security, diet diversity and the right to adequate food^a^.

	Food-plenty season (n = 422)			Food-poor season (n = 388)		
Question	Affected (n = 211)	Control (n = 211)	P value^b^	Affected (n = 191)	Control (n = 197)	P value^c^
In the past 30 days, instances when:						
(a) A household did not have sufficient food for more than 2 days						
Yes	107 (50.7)	103 (48.8)	0.77	125 (65.4)	89 (45.2)	0.000*
No	104 (49.3)	108 (51.2)		66 (34.6)	108 (54.8)	
(b) A household head felt the household was not eating food that was safe						
Yes	172 (81.5)	145 (68.7)	0.000*	174 (91.1)	130 (65.9)	0.000*
No	39 (18.5)	66 (31.3)		17 (8.9)	67 (34.1)	
(c) A household head felt the household was eating less nutritious food and could not do much about it						
Yes	153 (72.5)	113 (53.6)	0.000*	142 (74.3)	126 (63.9)	0.000*
No	58 (27.5)	98 (46.4)		49 (25.7)	71 (36.1)	
Does providing food for your household limit your ability to provide other amenities like health, water, housing, clothing and education?						
Yes	166 (78.7)	168 (79.6)	0.000*	125 (65.4)	110 (55.8)	0.000*
No	45 (21.3)	43 (20.4)		66 (34.6)	87 (44.2)	
Do you think landslides have affected your household’s food and nutrition security?						
Yes	152 (72.0)	133 (63.0)	0.004*	170 (89.0)	155 (78.6)	0.018*
No	59 (27.9)	78 (36.9)		21 (10.1)	42 (21.3)	
Are you aware about the principles of human rights of participation, accountability, non-discrimination and transparency?						
Yes	42 (19.9)	60 (28.4)	0.000*	38 (19.8)	57 (28.9)	0.000*
No	169 (80.1)	151 (71)		153 (80.2)	140 (71.1)	
Are you aware about the State obligations of respect, protect and fulfill						
Yes	18 (8.5)	28 (13.7)	0.000*	17 (8.9)	27 (13.7)	0.000*
No	193 (91.5)	183 (86.7)		174 (91.1)	170 (86.3)	
What would be the most important aspect for ensuring the right to adequate food among victims of landslide disasters?						
Cash hand-out	127 (60.2)	115 (54.5)	0.000*	164 (85.8)	124 (62.9)	0.000*
Resettlement land for agriculture	73 (34.6)	83 (39.3)		22 (11.5)	65 (32.9)	
Relief food	11 (5.2)	13 (6.2)		5 (2.6)	8 (4.1)	

^a^ Values are numbers (%).

^b^ P-value is for chi square test between affected and control during food-plenty season.

^c^ P-value is for chi square test between affected and control during food-poor season.

*Denotes significant association when P < 0.05.

Additionally, there were significant differences in responses between the affected and control households during both food seasons on the question regarding if a household head felt the household was eating less nutritious food and could not do much about it (p < 0.001 in both). A total of 72.5% of the affected compared to 53.6% of the control during the food-plenty and 74.3% of the affected compared to 64.9% of the control during the food-poor, reported that their households were eating less nutritious food, but could not do much about it (Table 4). Similarly, KIs expressed intake of less nutritious food among the affected communities. Specifically, reliance on low quality food e.g., dry tea and poor quality roasted banana with no sauce was reported to be consumed on several days by the affected communities (S1 Table).

Regarding if landslides affected the households’ food- and nutrition security (given a choice of yes or no), there were significant differences in responses between the affected and control households during both seasons (Table 4). A high proportion of both the affected (72.0%) and the control (63.0%) during the food-plenty season while 89.0% of the affected and 78.6% of the control during the food-poor season, reported that landslides affected the households’ food- and nutrition security. Moreover, a significantly higher proportion of the affected households reported that the provision/sourcing of food limited their ability to provide other amenities like health, water, housing, clothing and education during the food poor-poor season compared with the control households (55.8%) (Table 4).

KIs and FGDs further acknowledged that landslides affected the food and nutrition security and the RtAF of landslides victims. Landslide effects were linked to disruption of the social determinants of health (food, nutrition, water, education, sanitation, land and transport). Destruction of crops, water contamination and outbreak of epidemics like cholera immediately after landslides stood out as key issues among the KIs and FDGs (S1 Table).

Awareness about the principles of human rights (participation, accountability, non-discrimination and transparency) and the State obligations of respect, protect and fulfil was significantly lower among both the affected and the control at both seasons (p < 0.001 in all) (Table 3). Similarly, the discussions from FGD were shallow in relation to whether the human rights principles of participation, accountability, non-discrimination and transparency were taken into consideration during the response of public authorities to the disasters. This was due to low awareness about human rights including the principles of human rights among the FDG participants and KIs. Human rights were thought to be issues of the developed countries as pointed out by one FDG participant (S1 Table). However, some FDG participants interpreted topics about participation and non-discrimination in relation to decision making and distribution of relief food during disaster management. FDG participants noted that the elected leaders participated in decision making on their behalf and there was no discrimination of any case in relation to food distribution (S1 Table). Low awareness about the principles of accountability and transparency were also a challenge among the key informants who acknowledged not to be fully conversant with all the principles of human rights (S1 Table).

Concerning the obligation of the State to ensure that no Ugandan suffers from hunger and malnutrition even in times of disaster, KIs agreed that it was the government’s obligation to ensure that no Ugandan suffers from hunger and malnutrition even in times of disasters (S1 Table). The government’s obligations were linked to provision of relief food and creation of an enabling environment that allows non-state actors to participate in the disaster response processes to mitigate food insecurity and malnutrition.

**Fig 4:**
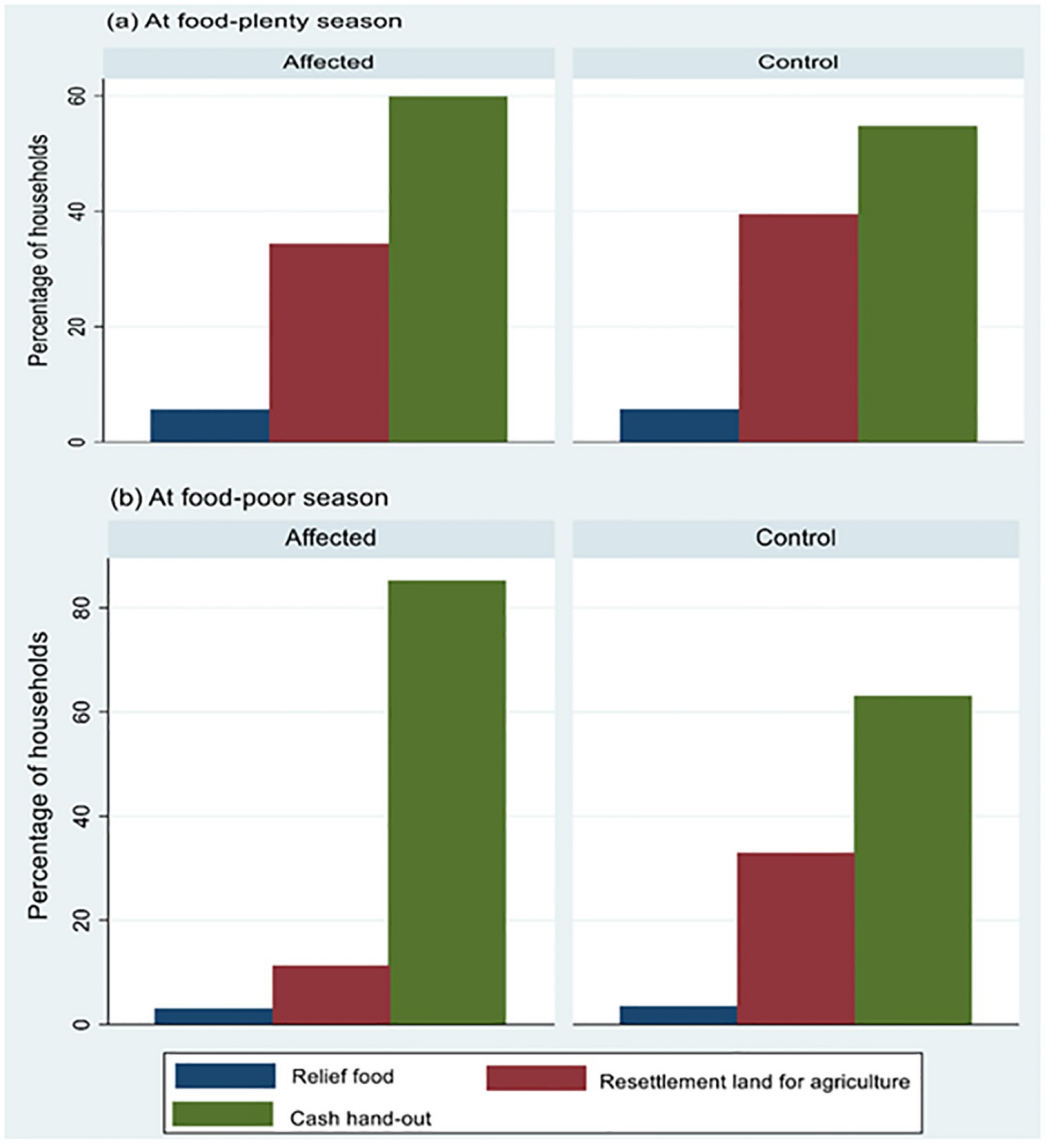
Most important aspect for ensuring the right to adequate food among households in the landslide-prone communities during: (a) food-plenty season (May-August) and (b) food-poor season (January-March).

When asked about the preferred means to ensure the RtAF of disaster victims among the three choices of: relief food, cash-hand out, or resettlement land for food production, the most preferred means to ensure the RtAF of disaster victims were cash hand-out followed by resettlement land for agriculture by both the affected and the controls during both seasons (Fig 4).

A difference in responses between the affected and control households was exhibited during both food seasons (p < 0.001in both) (Table 4). Equally, FGDs and KIs mentioned that provision of cash hand-outs as the most preferred means for ensuring the RtAF among landslide victims (S1 Table).

Regarding whether the disaster response in the study area was satisfactory; both the FGDs and KIs expressed lack of satisfaction about the disaster response in the study area. Relief food usually beans and posho (maize cornmeal) was cited as the main disaster response received from the government yet the landslide victims usually had other needs like shelter, clothing, safe water, cooking fuel and psycho-social support among others. The lack of variety in the relief food and inability to target the nutritional needs of vulnerable groups specifically the young children below 5 years stood out as a major issue (S1 Table).

On the issue of how the State should ensure the realization of the RtAF of landslide disaster prone communities, varied responses from FGDs and KIs included: sensitization of people about the RtAF, enforcement of existing policies, creation of an enabling environment for people to feed themselves in dignity and provision of adequate food in circumstances beyond peoples’ control (S1 Table).

## Discussion

The affected households presented relatively higher food insecurity and lower dietary diversity scores during both food seasons compared with the controls and the magnitude increased during the food poor season. This contradicts findings in our previous study [29], that found lower food insecurity and higher dietary diversity among the landslide affected communities in Bududa District. This contrast is possibly due to the massive and disastrous nature of the 2010 landslide disaster that gathered both national and international disaster response in terms of emergency interventions in areas of water, sanitation, hygiene, health promotion and relief food assistance among the landslide victims [29, 45, 46], hence the reduced food insecurity and higher dietary diversity. Consistent with our current findings, a recent study in Haiti found more food insecurity and poor dietary diversity among participants who were severely impacted by a hurricane compared to the less severely impacted participants [47]. In our setting, the relatively higher food insecurity and low levels of dietary diversity might be attributed to the long-term effects of landslide disasters and related shocks that led to prolonged deprivation of livelihoods and the means to secure an adequate and a diverse dietary among the affected households [3, 48].

Our study also found that the severity of food insecurity and lower dietary diversity among the affected households increased during the food-poor season. This is in line with studies from rural Southwest Uganda [49] and South Ethiopia [50] that reported increased food insecurity during the dry lean season compared to the food-plenty season. The food-poor season is characterized with lower food availability both on the farms and on the market, thus the affected probably faced both limited physical access to food on the farm and limited economic accessibility to food on the market due to low purchasing power. Household dietary diversity is a proxy indicator of a household’s economic access to a variety of foods [51]. This may suggest that landslide victims’ financial costs associated with acquisition of food for an adequate diet could have been threatened by lack of resources during the food-poor season. Equally, consumption of a lower diversified diet may indicate that the affected households’ diets were nutritionally inadequate. Prolonged intake of a nutritionally inadequate diet is linked to multiple micronutrient deficiencies that lead to impaired physical and cognitive development, poor physical growth and reduced work productivity which have negative macro-economic impact [52]. Poor diets also contribute to one in five adult deaths, through both insufficient intake of healthy foods and excess intake of unhealthy ones [48].

After controlling for socio-demographic covariates, our findings indicated that regardless of the food season, the disaster event was associated with both food-insecurity and dietary diversity, however the severity was more during the food-poor season and more among the affected households than the controls. Natural disasters are a leading cause of food insecurity as they affect all components of food security, reducing economic and physical access to food availability, utilization, and stability [53]. As such, persistent exposure to landslide disaster probably exposed the community to reduced food supply, and could have restricted access to safe and nutritious food, reduced quantity and quality of food consumed [2]. Moreover, the landslide affected community is located on steep mountainous terrain, restricting accessibility to market places for households to purchase a variety of food to complement their household diets. Increased availability and accessibility to markets usually conditions households to rely on market purchases to improve the diversity of household consumption [54].

The persistent exposure to disasters creates not only immediate effects, but also long-term effects. Landslides usually involve destruction of survival livelihoods, cause loss of human lives and damages to food crops, animals, houses and infrastructures such as schools, markets, health centers, bridges and roads, which directly or indirectly increase the landslide victims’ vulnerability to food insecurity. The widespread losses from landslides, which are beyond the landslide victims’ capacity to cope with their own resources, is thus not only short-term, but also long term. Therefore, exposing the victims to future food shortages will be manifested in both food seasons.

Primary education level was associated with both household food insecurity and low dietary diversity in terms of scores during both food seasons. Education is one of the determinants of household food security because of its association with economic status of a household [51, 55]. Wealthier households have the resources to purchase more and diverse food than poor households [51]. Less educated parents tend to have lower household income and higher poverty levels and hence have a low purchasing power for more nutritious and highly diversified foods. They may also have limited nutritional knowledge on how to meet health and nutritional needs for the household members.

Livelihood source was not an important factor associated with food security during the food-poor season. This is probably because the majority of the population in the study area is rural and depends mainly on rain-fed subsistence agriculture as a major source of livelihood [23, 35]. In rural subsistence agricultural settings, the food-poor season is characterized by intensive preparation of farm lands, depleted food stocks from the previous harvest and limited income-generating avenues [56, 57]. This leads to decreased availability and accessibility to food, both on the farms and on the markets due to lower crop production and higher food costs respectively.

The majority of both the affected and the control households answered in the affirmative when asked to the questions on the household not eating food that was safe and on the question of a household eating less nutritious food and could not do much about it. This indicates that a bigger proportion of the affected and control households’ diets were consuming nutritionally inadequate and unsafe food. Consumption of less nutritious and unsafe food may compromise the overall health and the nutritional status of landslide victims and thus further increasing their vulnerability to food insecurity and poverty related shocks and effects. Additionally, this contradicts paragraphs 10 and 11 of GC 12 that emphasizes the importance of assuring food safety and the perceived nonnutrient-based values attached to food and food consumption as crucial for the realization of the RtAF [3]. Also this may further delay the progress towards achieving SDG Target 2.1 of ensuring access to safe, nutritious and sufficient food for all people all year among the vulnerable victims of landslide disasters.

A considerable proportion of the households reported that the high expenses and economic demands on provision of food for their households limited their ability to provide other amenities like health, water, housing, clothing and education. Similarly FGDs and KIs cited landslides to affect sectors of food, health, water, education and transport among others. This reaffirms the interdependency, indivisibility and interrelatedness of humans rights [3]. Inability to achieve one human right, such as the right to adequate food, does affect the realization of other rights like in this case, the right to health [58–60]. This shows that households in Bududa District were accessing food in ways that were not sustainable and thus interfering with the enjoyment of other human rights. This is inconsistent with paragraphs 8 and 13 of the GC 12 that stresses that food should be accessible in ways that are sustainable such that the attainment of other basic needs are not threatened or compromised as a core condition for the realization of the right to adequate food [3]. It may also be plausible to argue that, the households were struggling to put food on the table and in doing so, they compromised or constrained the attainment of other basic needs like safe water, health and housing.

Cash-handout stood out as the most preferred aspect for ensuring the RtAF among the affected and control households during both food seasons. This contradicts our previous findings [61] where both the affected and control households preferred the provision of land for food production as the outstanding choice to ensure the RtAF of disaster victims. This is probably linked to previous findings in the same area which showed that the relief food in the area was of limited variety mostly dominated by dry rations of maize flour and beans, often less preferred and less desirable [61]. Similarly, this is possibly because the landslide victims were previously resettled in a different district on land with lack of land ownership and not sensitive to the *“Bamasaba”* culture and food security needs. It is plausible that the provision of cash presents the landslide victims with the opportunity to be resettled to safer areas of their choice and on land with full land ownership rights and with favorable and familiar factors such as high soil fertility, geographical location similar to Bududa District and sensitive to the *“Bamasaba”* culture including culturally acceptable foods. Similarly, provision of cash is thought to be quicker compared to construction of houses for the landslide victims as noted by the State Minister in charge of disaster preparedness management in Uganda [62].

Our findings also indicated low awareness about the RtAF, State obligations and principles of human rights among the study participants. This corroborates findings in Uganda that found low knowledge and low awareness on the RtAF and related State obligations among duty bearers [63, 64] and rights-holders [61]. Knowledge and awareness about the RtAF by duty-bearers and rights-holders is an essential pre-condition for the realization of the RtAF. This situation of limited awareness of human rights and the right to adequate food in particular by the key State actors narrows the possibilities of pursuing for remedies and recourse mechanisms in the case of violations. Whereas rights-holders may be deprived of this human right without knowing it [43], they need to know whom to hold accountable and to whom they should direct complaints in case of violations of their RtAF.

A major strength of our current study is the longitudinal cohort design that allowed for an account of possible seasonal variations in food insecurity and dietary diversity among victims of landslide disaster. We employed a mixed methods approach to add credibility and depth to the findings as recommended in the human rights research approach [37]. Study limitations included the possibility of bias in socio-economic and demographic variables, and we do not have data on actual food intake, body composition or biomarkers of nutrient intake. Moreover, the landslide affected sub-county may have differed from the control (neighboring) sub-county in other aspects than just landslide. Floods were also experienced during the study period, and possibly they may have affected the food and nutrition outcomes of the study participants.

We conclude by re-echoring that, this study provides evidence of the impact of seasonal variations on food insecurity and dietary diversity among the rural vulnerable populations distressed with landslide disasters in Uganda. Whereas the severity of food insecurity and low dietary diversity were more pronounced among the affected households than the controls during both food-seasons, the right to adequate food of landslide victims was not sufficiently realized. Therefore, underlying determinants of food insecurity, dietary and the RtAF among poor rural landslide prone households should be addressed in an integral manner. The Uganda National Development Plan III 2020/21-2024/25 and its specific programs which are crucial for food and nutrition security, should give greater attention to the serious and growing problem of landslides. Strengthening and expanding the social protection programs to alleviate landslide victim’s vulnerability to food insecurity in the face of landslides is key if we are to achieve “zero hunger” by 2030 and the right to adequate food for all. Policy actions that promote landslide victims’ accessibility to and ownership of land in risk-free areas are important. Similarly, policies that promote nutrition-sensitive agricultural production, diet diversification and robust legally appropriated and reliable disaster-specific public social safety nets such as unconditional cash transfers are of essence. In the long-term, education and income diversification program interventions need to be integrated into disaster recovery programs since they are central in enhancing the resilience of rural livelihoods to shocks and stressors affecting the food system.

## Supporting information

S1 Table Themes and quotes from focus group discussants and key informants. (DOCX)

S1 Checklist STROBE statement—Checklist of items that should be included in reports of *cohort studies*. (PDF)

S1 Questionnaire Inclusivity in global research. (PDF)
