# Supplementary material for: Food insecurity, dietary diversity and the right to adequate food among households in landslide-prone communities in Eastern Uganda: A cohort study
Source: PLoS One. 2023 Apr 13;18(4):e0283078. doi: 10.1371/journal.pone.0283078 (PMC10101418; doi:10.1371/journal.pone.0283078)
Supplement: S1 Table — (DOCX) [file pone.0283078.s001.docx]

**S1 Table. Themes and quotes from Focus the Group Discussants and Key Informants**

| **Question** | **Themes** | **Quotes** |
| --- | --- | --- |
| What is the situation of food and nutrition security in the study area? | Food and nutrition insecurity | “*Generally, the food and nutrition security in Bududa District is bad due to the persistent landslides that affect so many communities…”* (KI-1*)*  *“The situation is not good at all; our people here do not have enough food to eat. Sometimes they rely on only a few varieties of foods”* (KI-3)    *“Bududa District is a food basket in this region with nutritious foods, however due to the persistent landslides, the food and nutrition security has become poor”* (KI-8)  *“ …the food and nutrition situation is very bad…especially to us who have suffered from landslides, we hardly have enough food to eat and to feed our children”* (FGD-2 Affected)  *“ …because of persistent lack of enough food and money, many of our people are consuming maize flour and beans which are insect-infested and with a bad smell and taste throughout the food seasons*” (KI-2) |
|  | Undesirable diet diversity | *“We mainly consume beans and posho almost every day… we do not have money to buy other foods like meat…” and “the situation becomes worse during the dry season* *where you can hardly get any vegetable in the garden…”* (FGD-4 Affected)  *“Our people rely depend on a monotonous diet all every day and the situation is tougher during the dry season ”* (KI-1) |
| Where? | Landslide affected community | *“Immediately after landslides have occurred, the food security is very bad among the landslide affected communities”* (FGD-1 Control)  *“Our people in the landslide affected communities are more affected than the people in the non-landslide affected communities* *and the situation is worse during the dry season”* (KI-9) |
| Who are most affected? | Vulnerable groups | “*The most affected are generally young children below 5 years, pregnant and lactating mothers, the elderly and the sick”* (FGD-2 Affected)  *“The young children are affected especially when the family head who was a bread winner dies due to landslides”* (KI-10)  *“ The young children are the most affected”* (FGD-8 Control) |
| When? | Seasonality | *“The food situation is bad since the area is a hot spot for landslides. However, it is worse immediately after a landslide and during the dry season”* (KI-3)  *“It is usually poor during the dry season when there’s hardly any food in the gardens and the food in the market is usually very expensive”* (KI-7).  *“It is somewhat better during the harvest season as we have food to eat and we can sell some food to the market to buy another food like meat”* (FGD-8)  *“During the dry season, most of the crops dry off including vegetables…there is scarcity of food in the dry season””…and it is even worse when we do not have money for buying food”* (FGD-10 Affected) |
| Whether landslides affected the food and nutrition security and RtAF of landslides victims | Right to adequate food dimensions | *“Yes, landslides have a very big impact on the food security of our people”* (KI-2)  *“Yes, landslides seriously affect the type of food we eat…; landslides destroy our crops and kill the few livestock we have. This makes us to have no food or limited food for consumption”* (FGD-16 Affected)  *“When a landslide occurs, food is destroyed, the animals are killed, roads are cut off and people fail to access their gardens”* (KI-10)  *“ “Landslides destroy the crops, animals, poultry which families depend on”* (KI-9).  *“Animals like cattle are killed, therefore no more milk for the children”*(KI-6)  *“Water gets contaminated and we have to walk long distances in search of water for drinking and home use”* (FGD-7 Affected group)  *“Vegetables are washed away, and we remain with no vegetables to cook”* (FGD-4 Affected)  “*Landslides usually are followed with some epidemics like cholera that mainly affect our young children”* (KI-1)  *“Our people consume poor quality and quantity of food since most of the food gets destroyed during landslides”… and many of our people can hardly consume 4 meals per day and the situation is worse during the dry season”* ( KI-3)  “*When landslides occur, almost all sectors are affected...food is destroyed, water systems and sources get contaminated, transport is cut off, hence no access to health centers by both the service providers and the local natives and there is limited accessibility to land for food production…”* (KI-8) |
| Whether the disaster response in the study area is satisfactory | Right to adequate food dimensions | “*No, it is not. Satisfactory. Usually the Office of the Prime Minister sends in relief food which is mainly beans and posho”…yet the disaster affected victims usually face many more problems ranging from lack of shelter, clothing, cooking fuel and psycho-social support among others…”* (KI-1)  *“The relief food does not take into account the nutritional requirements of specific vulnerable age groups like the young children”* (KI-4)  *“Disaster response is improving, it is not like before, at least we now have emergency capacity like tents to handle emergencies due to disasters”* (KI-3)  *“We are all given the same type of food irrespective of whether you have a young child or not!”* (FGD-7 Affected) |
| Whether the human rights principles of participation, accountability, non-discrimination and transparency are taken into consideration during the response of public authorities to the disasters | Right to adequate food dimensions | *“ We are not aware about those things of human rights, the people in the big offices are the ones who know them”* (FGD-13 Control)  *“ Our leaders sometimes meet with the government to represent our issues concerning human rights”* (FGD-5 Affected)  *“Accountability and transparency are not well understood, even us the leaders of this area are not fully conversant with all the principles of human rights”* (KI-9)  *“Our people partly participate in the decision making through their elected leadership”* (KI-5)  *“Equality and non-discrimination are upheld at all stages during distribution of relief food, … when food is being distributed, no one is discriminated* *on the basis of race, colour, ethnicity, gender, age, language, religion, political or other opinion, national, social or geographical origin, disability”* (KI-2 )  *“Human dignity is ensured ,government distributes food that is fit for human consumption”* (KI-1)  *“Human rights are issues of the developed countries…; they cannot work here in our poor country...”* (FGD- 12 Affected) |
| What is your perception on the fact that it is the obligation of the State to ensure that no Ugandan suffer from hunger and malnutrition even in times of disaster? | Right to adequate food dimensions | *“Yes it the government’s obligation, to ensure that no Ugandan suffers from hunger and malnutrition even in times of disaster”* (KI-3)  *“We are not aware of state obligations; however we know that if we are faced with landslides, the government is supposed to help us with food and shelter…”* (FGD-2 Affected)  *“Our government provides relief food during disasters… and has allowed Civil Society Organisations and Non-Government organisations to participate in the disaster management processes to fight food insecurity and malnutrition”* (KI-10)  *“Government should put up and enforce strong policies that will prevent people from suffering from hunger and malnutrition”* (KI-3) |
| How the State should ensure the realization of the right to adequate food of landslide disaster prone communities | Right to adequate food dimensions | *“Government should sensitize the people about these rights, so that people can demand for them from the people with authority in the respective government institutions”* (KI-6)  *“Government should educate us about human rights through our local radio stations and our local leaders”* (FGD-10 Control)  *“Government should create new policies and strictly enforce the existing policies that are linked to food and nutrition security ”* (KI-2)  *“ In times of disasters, government should provide a well-diversified and balanced diet to the disaster victims”* (KI-4)  *“ Government should create an enabling environment that ensures that every Ugandan feeds himself in dignity”* (KI-3) |
| The most preferred means to ensure the right to adequate food of disaster victims | Right to adequate food dimensions | *“Cash hand-out is most preferred aspect for ensuring the right to adequate food because it gives the victims liberty to buy land and food of their choice”* (KI-1)  *“Relief food is often of limited variety and of poor quality and some landslide victims sell off the given relief food”* (KI-5)  *“Cash handout is the best option since it comes with the freedom for one to buy safe land of his or her choice”* (FGD-14 Affected)  *“Cash handout is the best…for people to buy their own food”* (FDG-13 Control) |

Abbreviations: KI- Key informant; FGD- Focus Group Discussion; RtAF- Right to adequate food
